# Drivers of Patients’ Behavioral Intention toward Public and Private Clinics’ Services

**DOI:** 10.3390/healthcare11162336

**Published:** 2023-08-18

**Authors:** Zohra Ghali, Karim Garrouch, Abdulrahman Aljasser

**Affiliations:** 1 Department of Business Administration, College of Administrative and Financial Sciences, Saudi Electronic University, Riyadh 93499, Saudi Arabia; kgarrouch@seu.edu.sa; 2 Master in Business Administration, Saudi Electronic University, Riyadh 93499, Saudi Arabia; a.s.a.aljasser@gmail.com

**Keywords:** service quality, reputation, trust, service value, behavioral intention, healthcare service, patient

## Abstract

In an era of growing competition in the healthcare market, adopting a patient-centered approach is mandatory for the survival and growth of any public or private hospital. This requires a better understanding of patients’ behavior and an increased focus on satisfying their needs and expectations. This paper was developed in this context and aims to study the main drivers of patients’ behavioral intentions. A conceptual model was proposed, highlighting the linkages between service quality, doctors’ reputation, patients’ trust, service value, and patients’ behavioral intentions. To examine the different research hypotheses, a quantitative study including 242 patients was conducted in Saudi Arabia using the convenience sampling method. The smart PLS approach was used to test the measurement and structural models. The findings indicated that trust and service value positively affected patients’ behavioral intentions. Trust in the healthcare provider was positively affected by two dimensions of service quality: healthcare provider concern and physician concern. Trust in doctors was found to be positively related to the reputation of the doctor. Service value was positively influenced by the convenience of the healthcare process, healthcare provider concerns, and doctors’ reputations. This study is original because it is among the few studies that investigate patients’ behavioral intentions toward healthcare services in a developing country (Saudi Arabia). Furthermore, it is among the rare studies to examine the role of doctors’ reputations in service values. The findings would offer meaningful implications for practitioners in the healthcare market for maintaining relationships with their patients.

## 1. Introduction

The healthcare industry is currently undergoing substantial changes and challenges because of the increased competition among healthcare providers [1,2,3]. Healthcare providers must combine the traditional medical approach, which emphasizes the effectiveness and efficacy of healthcare outcomes from the provider’s perspective [4,5,6], with the patient-centered principle, which emphasizes patients’ concerns and interests [7], to create and maintain a competitive advantage. From this perspective, healthcare service providers are required to attract patients and maintain a durable relationship with them [8,9]. The existing literature offers multiple research studies on the behavioral intention of customers over time; however, little focus has been directed to patients’ behavioral intentions toward public and private clinics. Indeed, the literature proposes scattered cues and variables explaining patients’ perception of service value and their trust towards healthcare stakeholders, all triggering behavioral intentions. Healthcare service quality and value are vital concepts to attract patients and behaviors [10,11]. In this regard, value-based healthcare is a perspective that still gathers impetus, which provides better value for healthcare services [12,13,14,15].

Choi et al. [4] are among the pioneers who focused on quality, value, and behavior in the context of healthcare services and from a marketing perspective. Their approach is built on three focal variables: service quality, service value, and behavioral intentions. Since then, the concept of value has not received sufficient attention as a factor explaining patients’ intentions toward public and private hospitals. In addition, there is a lack of studies that verify its antecedents. Thus, the first research gap that this study aims to address is to highlight the main drivers of patients’ behavioral intentions toward public and -private hospitals. These drivers are trust in doctors, trust in healthcare service providers, and service value. In addition, Choi et al. [4] verified the direct impact of the entire quality concept on the perception of service value. Their operational definition of quality was mainly based on four dimensions: the convenience of the healthcare process, the healthcare provider concern, the physician concern, and tangibles. However, they verified the impact of the aggregated measure of quality, rather than the direct impact of each dimension. Thus, the second contribution of this paper is to address the research gap which is the lack of studies investigating the direct association between the dimensions of service quality and the reputation of doctors, from one side, and the patient’s trust (in doctors and service providers) and the perceived value, from the other side.

The main objective of this study is to develop and verify a conceptual model as an extension of the Choi et al. [4] approach, which highlights the main determinants of a patient’s behavioral intention. The idea is founded in the Means-End Chain Theory (MECT), which focuses on understanding how service attributes contribute to customer value, leading to positive behaviors toward the service provider [16]. In this theory, value is considered the final goal among a hierarchy of goals triggering a behavior, while benefits are considered sub-goals that may subordinate such values. In the service context, quality has been proposed as an antecedent of perceived value which leads to behavioral intentions [17]. Based on the work of Choi et al. [4], we used three healthcare service quality dimensions as predictors of perceived value: the convenience of the care process, the healthcare provider concern, and the physician concern. In addition, we used Spence’s signaling theory [18] to extend Choi et al.’s [4] framework by integrating trust as a consequence of quality dimensions and reputation. Thus, we focused particularly on trust in the service provider, trust in the doctor, and service value, which is determined by doctors’ reputations and three different quality dimensions: the convenience of the care process, the healthcare provider concern, and the physician concern. This paper is interesting from both theoretical and practical perspectives. Theoretically, this study contributes to the existing literature by examining the importance of both facets of trust and value as important predictors of behavioral intentions toward a healthcare institution. In addition, the verification of the impact of each element of healthcare service quality constitutes another contribution of this paper, as it sheds light on the importance of the facets of healthcare service quality. Practically, this study provides significant recommendations and insightful implications for practitioners in the healthcare sector to improve the behavioral intentions of their patients in both public and private hospitals.

The remainder of this paper is structured as follows: the second section presents the literature review, where the focus is on the different variables of the proposed conceptual model as well as the relationships between them. The third section describes the research methodology. The fourth section presents the results and verifies the research hypotheses. The fifth section discusses the results of this study. The sixth section presents the implications of the research, limitations, and future pathways, and the final section presents the conclusion.

## 2. Conceptual Framework and Research Hypotheses

### 2.1. Behavioral Intention toward Healthcare Service Providers, Patients’ Trust, and Service Value

Healthcare service refers to the service that contributes to the improvement in a person’s health through preventing, diagnosing, treating, rehabilitating, and/or prescribing the cure of diseases and other mental and physical impairments in people [19,20]. It is not a product manufactured by healthcare institutions, but it is a service cocreated by professionals (physicians, nurses, medical staff, etc.) on the one hand, and patients looking to restore or maintain good health for themselves or their families on another hand [21].

Healthcare services providers include different institutions and organizations supplying services to patients within a defined financial and regulatory framework, like private and public hospitals. As this market has witnessed significant competition these last decades, understanding patients’ behavior becomes interesting to customize the offered healthcare services and build robust relationships with them. Then, understanding the main drivers of patients’ behavioral intentions becomes interesting for any healthcare service provider.

Behavioral intention is defined as a certain likelihood of engaging in behavior [22]. It encompasses the intention to use, which is the extent to which the consumer formulates a will to perform a specific behavior. It also includes the willingness to pay more for a service or a good [22]. The theory of planned behavior considers that this variable is formed based on a favorable customer’s evaluation regarding a service or a good [23]. In the same context, Baker and Crompton [24] considered the behavioral intentions of a patient as a willingness to maintain a relationship with the same service provider. It is expressed by the revisit or return intention of a customer. It constitutes a critical indicator of service providers’ achievement, as long as it is a crucial determinant of loyalty toward a particular service provider [7,25]. In the healthcare industry, the behavioral intentions of a patient encompass the willingness to revisit the hospital for more treatment and the recommendation of that hospital to others [26]. Several scholars [26,27] have stated that behavioral intention should be used as a critical indicator of the success of service enterprises. The marketing literature has distinguished several motives for the behavioral intention of patients. In this study, we focus on trust in service providers, trust in doctors, and service value.

The concept of trust was introduced by Parasuraman et al. [28], who consider it the most critical factor for the success of any durable relationship between service providers and customers. Morgan and Hunt [29] stated that trust is among the primordial attributes (with commitment and shared values) that prevent opportunism in relationships and favor loyalty. Based on Mayer et al.’s [30] conceptualization, we define it as an individual’s willingness to be “vulnerable” to the provider’s actions. This means that trust is a critical stabilizer of relationships. In commercial contexts, consumers who trust a provider have feelings of safety, assurance, and confidentiality regarding the service transaction [14]. In healthcare services, patients may build trust in a provider from their first contact. They then maintain and strengthen it over time through the repetition of transactions. This is because trust reduces uncertainty about future transactions and ensures greater stability in relationships between customers and service providers [31].

According to Porter [32], creating value for patients is a mandatory goal of healthcare delivery. He defined it as an evaluation of the ratio between health outcomes and spent money. It requires all actors in the medical system to make the patient the center of their interests. This is because all partners (suppliers, payers, providers, etc.) can profit from the increase in service value [10]. Consequently, the economic sustainability of the healthcare system has improved [32,33]. Service value is an indicator of the performance of the healthcare industry [4]. Building rigorous, disciplined, and growing services is the best way to develop a healthcare system. Therefore, Normann [34] (p. 124) argued that it is crucial to create value for the sake of supporting “health-giving processes” rather than concentrating only on “curing” tasks. The value should be created in different steps of interaction with the healthcare provider. In the first stage, patients attempt to make an appointment with the healthcare service provider. In this early stage, the value process is initiated. For patients, it should be possible to book an appointment as soon as possible. This is considered service accessibility, which can create value for healthcare services [35]. The next step is to contact a doctor. This is the step when the service is created through dialogue and activities between customers and doctors. The third step is to make an appropriate diagnosis in the shortest possible period, and the last step is the continuity of the relationship with the medical staff. The more patients are satisfied with the service through the different stages of its creation, the more they maintain their relationship with the healthcare provider.

### 2.2. Service Quality: Drivers of Service Value and Trust

Service quality refers to the customer’s perception of service excellence and its superiority [28]. It has become a major concern for healthcare institutions around the world [36]. The growing focus on patients’ needs and expectations has led scholars to investigate the service quality dimensions and how they can be measured [37,38]. Various approaches have been used to measure service quality in the healthcare sector, including the dyadic approach. The latter considered that the perception of the quality of both seekers (patients) and service providers (hospitals) is critical to improving the relationships between partners and enhancing performance in the process of healthcare institutions [15]. To assess hospitals’ service quality (HSQ), this approach proposes a scale that focuses on measuring both knowledge and perception gaps [38]. This allows practitioners to measure, on the one hand, the gap between service providers’ perceptions of patients’ expectations and their actual needs and expectations, which translates into the knowledge gap. On the other hand, this approach proposes a scale that measures the gap between what is delivered by hospitals and what is received by patients, which translates into the perception gap. HSQ is a multidimensional concept [5,37,38]. In the context of this study, three dimensions are highlighted: convenience of the healthcare process, healthcare provider concern, and physician concern.

### 2.3. The Convenience of the Healthcare Process

With changes in individuals’ references and the growth in information technologies, service convenience has received increasing attention [39]. In the healthcare industry, patients request more convenience in the delivery and administration of care, such as e-health services, personal health information via special devices (smartphones, mobiles, tablets), and e-medical records, which replace paper in doctors’ offices [39]. In other words, with the advancement in IT, convenience should shift from a goods-centered perspective to a service-oriented view.

According to Maarse and Todd [40], convenience is a critical factor for the success of any service. In the healthcare context, Zainuddin et al. [13] defined convenience as the simplification of the wanted behavior by implementing appropriate processes and structures. This encompasses the convenience of the service center’s location, advantageous facilities, namely parking spaces, and thoughtful services. Hence, patients require more than good care [41]. They also request ease, short waiting times, and good hospitality from the support staff. In other words, these patients need not only good healthcare but also a pleasant experience. According to Masterson [42], in the American market, a recent Kaufman Hall survey including 200 hospitals and healthcare executives found that convenience and access to care are the most critical factors in choosing a healthcare provider. Furthermore, almost ninety percent (90%) of the respondents expressed a need to improve their hospitality experience.

Convenience is a dimension of healthcare service quality [4] which can provide value and consumer delight to healthcare services. Hence, providing more convenience for patients is a critical determinant of service value and a primordial factor in building the trust of patients, as confirmed by several previous studies [43,44]. Prior studies argue that trust in hospitals is associated with trust in a physician [45], patient experience with the healthcare facility [46], and healthcare quality supervision [47]. Based on the above discussion, the following hypotheses were formulated:

**H1a.**  *The convenience of the healthcare process would significantly influence the trust in healthcare providers.*

**H1b.**  *The convenience of the healthcare process is positively related to the service value of healthcare providers.*

### 2.4. Healthcare Provider Concern

The overall perception of service quality is an outcome of an evaluation based on a comparison between one’s expectations and experienced benefits [48]. Indeed, the service quality provided by healthcare actors is assessed in terms of outcomes and benefits [4,34]. Indeed, patients require high-quality services, safety, convenience, and patient-centeredness [32]. Achieving high service value should become the primary role of all healthcare service providers. Several scholars have argued that quality is a predictor of trust [4,27]. The elaboration likelihood theory focuses on trust and explains it using two types of factors: primary and secondary. Quality dimensions are considered the primary determinants of trust [49].

Healthcare provider concerns include beneficial elements related to healthcare services, such as nurses’ friendliness, a good explanation of the medication process, and the belief that the provider has genuine care [4]. These elements can be considered benefits that compensate for the perceived sacrifices made to be under the control of that provider.

Knowing that, by definition, perceived value is a ratio between benefits and sacrifices [50], healthcare providers’ concerns may be considered a motive for perceived value. Accordingly, the following hypotheses were formulated:

**H2a.**  *Healthcare provider concern is positively related to trust in service providers.*

**H2b.**  *Healthcare provider concern is positively related to service value.*

### 2.5. Physician Concern

As it is known, the primary purpose of medicine is to care for sick people and fulfill patients’ needs [51]. Physicians, as medical doctors, play a central role in maintaining, promoting, and restoring the health of people by diagnosing and treating patients’ diseases and injuries [52]. Given the importance of their role in public health, physicians are ranked above many other professions and are expected to be honest and ethical [53].

Several scholars have confirmed that physicians’ roles have been developing and becoming preventive and social, rather than curative and individual [54]. Then, the doctor–patient relationship is based on an implicit social contract [53]. Such a contract requires, according to the agency theory, physicians to provide healthcare services on behalf of their institutions, including entrusting decision making about the patient’s diagnosis and treatment [55]. This theory was originally built by Arrow [56], who was the first to address the issue of asymmetric information in healthcare, mostly in health insurance services.

Based on agency theory, there is an imbalance of healthcare knowledge between physicians and their patients, conducting to the delegation of decision-making authority because of patients’ lack of knowledge regarding the diagnosis of diseases and their appropriate medication [57]. In this context, Major [58] revealed that despite patients being highly informed about symptoms, diagnosis, and even treatment nowadays due to easy access to health information via the Internet, they still do not have the competence of medical doctors to know how to analyze and treat symptoms and diseases. Therefore, physician concern and expertise play a vital role in ensuring the patient and building their trust in doctors, as well as healthcare providers [59]. Such physician concern constitutes, according to Choi et al. [4], an important dimension of healthcare service quality. Dobrzykowski and McFadden [57] argued that a strong and close relationship between patient and physician may drive patients’ trust in physicians and even in healthcare institutions. Heggins et al. [59] pointed out the vital role played by the efficient service of physicians in the implementation of patients’ trust in healthcare institutions. In the same context, Maarse and Jeurissen [40] show that trust in healthcare service providers is related to people’s comprehension of the process of the healthcare system, the financial motives of physicians, and providers’ behavior. Based on this discussion, the following hypotheses were formulated:

**H3a.**  *Physician concern is positively related to trust in healthcare service providers.*

**H3b.**  *Physician concern is positively related to service value.*

### 2.6. Reputation of Doctors

Einwiller [60] defined a person’s reputation as a third-party experience with a relationship partner. Reputation has a key role because it is a way to cope with the uncertainty of a service outcome. It is addressed based on the customer’s prior experiences and those communicated by others to reduce possible risks [61].

Reputation has been considered a signal supporting that the transaction and service will not be risky. Indeed, the signaling theory of Spence [18] has been used to justify that the reputation associated with the provider enhances trustworthiness. Reputation transmits information about the service and the service provided, especially in the case of the first use of the service [62]. This applies to healthcare and medical services. The link between value and reputation has been ignored by the prior literature. It has been rarely correlated, only in a few studies [63]. Indeed, these authors considered the impact of reputation, perceived value, and trust as factors explaining customer retention, and as variables that can be correlated. Kadhim et al. [64] consider reputation a premise for trust in a service provider because it stems from the beliefs or estimations about the quality of the service or the character and standing of the service provider. Thus, reputation is associated with an overall evaluation of the character of the service provider and their capacity to constantly satisfy the market’s needs.

In the healthcare industry, a strong reputation is a major asset for doctors. A doctor’s reputation is determined by the patients’ reception of direct and indirect experiences and information and by doctors’ achievements. Doctors receive several patients every day, each of whom has a particular set of attributes that justify their choice. Among these attributes, we list competent, qualified, capable, rigorous, good, trustworthy, reputable, believable, reliable, serious, or experienced as only a few attributes that make doctors known to their patients and have a strong positive reputation. According to Torres et al. [65] (p. 186), a doctor’s reputation plays a fundamental role in how patients gain trust in their healthcare service providers. In the same vein, Mechnic and Meyer [46] argued that doctors who have a good reputation are recommended by friends and families of patients. Thus, trust is stimulated by a good reputation. Based on what is said in this paragraph, the following hypotheses were formulated:

**H4a.**  *A doctor’s reputation is positively related to service value.*

**H4b.**  *A doctor’s reputation is positively related to patients’ trust in doctors.*

### 2.7. Drivers of Patients’ Behavioral Intention: Patients’ Trust and Service Value

#### 2.7.1. Patients’ Trust

Patient trust in a healthcare provider is defined as an acceptance of vulnerability in circumstances where this patient (the trustor) believes that the healthcare organization or another person (the doctor of the organization) will care for their interests [7,66,67]. Several studies have found a positive correlation between trust and customers’ willingness to purchase [51]. Hence, these authors defined trust in service providers as the “*willingness to be vulnerable*”. According to Zheng et al. [68] and Rahman et al. [67], trust can strengthen customers’ behavioral intentions toward a service provider. Kim et al. [69] verified the impact of trust on the value and well-being in the case of online travel agencies. In the context of medical services, Ngobo [70] argued that when healthcare providers fail to understand the expectations of patients and satisfy their needs in terms of reliability, integrity, and quality of services, the intention to continue their relationship with them becomes risky. Thus, the more trust the patient has in healthcare providers, the more they intend to maintain their relationship with them. Based on what is said in this paragraph, the following hypothesis was formulated:

**H5a.**  *Trust in healthcare services is positively related to patients’ behavioral intentions.*

Moreover, trust appears to be primordial in doctor–patient relationships. The more patients have trust in their doctors, the more they follow doctors’ treatment recommendations and advice [65,68]. In the same vein, Nordgren and Fridlund [71] stated that trust in doctors is a cornerstone of maintaining relationships with medical institutions. The prompt changes in the healthcare system make trust in physicians a mandatory determinant for maintaining the relationship with them [54]. Accordingly, the following hypothesis was formulated:

**H5b.**  *Trust in doctors is positively related to patients’ behavioral intentions.*

#### 2.7.2. Service Value

Service value is always considered a determinant of well-being, customer behavior, and an organization’s competitive advantage [4,69,72]. Globally, doctors and managers of hospitals face challenges in healthcare service value perceptions [73]. Therefore, the improvement in services constitutes a serious challenge for service providers [4]. It has been argued that studying healthcare consumer value is crucial for healthcare providers because of contemporary supply pressures and the evolution of consumer needs [74]. Nowadays, owing to changes in patients’ preferences and behaviors, it is critical to improve the set of services provided. Fornell et al. [75] confirmed this positive correlation between service value and behavioral intention.

**H6.**  *Service value is positively related to patients’ behavioral intention.*

**Figure 1:**
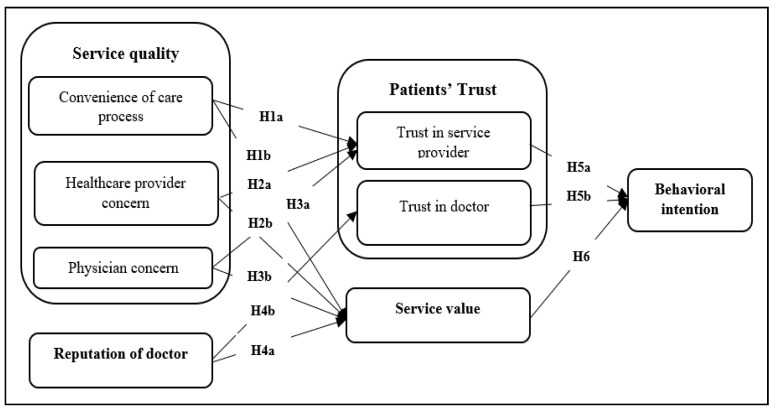
Conceptual framework.

Based on the above literature review, a conceptual model was proposed, as shown in Figure 1.

## 3. Methods and Materials

### 3.1. Data Collection and Sample

To test the hypotheses, a survey was conducted targeting patients seeking healthcare services at different private and public hospitals in the Kingdom of Saudi Arabia. Owing to the unavailability of a sampling frame for patients visiting all private and public hospitals in this country, non-probabilistic convenience sampling was adopted. However, we attempted to obtain users of health services by cutting across gender, age, and place. To obtain the required number from the population, the survey was created in Google Forms and then shared on social media groups, namely WhatsApp groups. Two former MBA students working in the domain of healthcare administration helped to identify the group on which the survey link was posted. The survey questions were in both Arabic and English. The number of responses received was 263. However, only 242 responses were considered because the others included the same responses (strongly agree) for all questions. The sample considered encompassed 51.6% men and 48.4% women. Over 8% of respondents were aged from 18 to 25 years old, more than 72% of respondents were aged from 26 to 40, and the others were aged more than 40 years old. Further, 62% declared they received health treatment in private institutions, while 38% used health services in public institutions. Over 90% of respondents were Saudi; the remaining were non-Saudi. The authors attempted to make the percentage of expatriates around 30%, which is the actual percentage in the last available statistics [76]. However, the availability of patients willing to answer the survey was a constraint and the acceptance rate was insufficient. This proportion was only around 9.5% of the sample.

The distribution of the sample base on demographics is shown in Table 1 below.

### 3.2. Measurement Scales

All the items were measured on a five-point Likert scale. The convenience of the healthcare process, healthcare providers’ concerns, and behavioral intentions were measured using the scale of Choi et al. [4]. Service value was measured based on Zeithaml [50]. Reputation was measured using the scale of Torres et al. [65] and trust toward the provider/physician was measured using the scale of Zheng et al. [68]. The measurement scale items are available in Appendix A Table A1.

The language of the items was either English or Arabic. To create the Arabic items, we translated the initial items using a back-to-back translation by two marketing researchers. An expert and researcher in English–Arabic translation helped ensure that the language and wordings were properly translated and interfered with adjusting the items whose structure after the back-to-back translation was different from the initial structure.

The SEM partial least squares method was used in SmartPLS version 3.2.7. This statistical tool is efficient when the sample size is small, even when the tested model is complex [77]. The SPSS24 was also used as a first step to explore the dimensionality and reliability of the scales.

## 4. Results

### 4.1. Measurement Model

The reliability of the measurements was assessed using two indicators: Cronbach’s alpha and composite reliability [78]. The scale’s composite reliability (CR) ranged from 0.861 to 0.944, thereby satisfying the recommended threshold of 0.7 [78]. The values of Cronbach’s alpha are all satisfactory, as they are superior to the recommended cutoff of 0.7 [79]. Regarding convergent validity, the values of the average variance extracted are all greater than 0.5, as suggested by Fornell and Larcker [78]. The reliability and convergent validity results are summarized in Table 2.

The cross-loading matrix shown in Appendix A Table A2 was used to verify discriminant validity. This shows that all items have higher load values with their constructs compared to their values on other constructs. This indicated that the discriminant validity of the item was acceptable.

### 4.2. Structural Model’s Predictive Relevance

#### 4.2.1. The Productiveness of the Model

The productiveness of the model was assessed via the dependent variable’s squared multiple correlations (R2), the test of predictive relevance of Stone–Geisser (Q2), and the effect size (Cohen’s f2). The latter measures R2 changes and shows the extent to which the independent constructs have an impact size [80]. This effect size is large if f2 is higher than 0.35, and trivial if less than 0.02. Accordingly, the two paths have trivial size effects: the effect of the convenience of the care process on trust toward the provider and the impact of physician concern on service value. The only path with a large size effect is that between a doctor’s reputation and trust. The remaining paths have a small effect size, as they have values lower than 0.15 [80]. The results are presented in Table 3.

The R^2^ values range between 0.374 and 0.558, which shows that a satisfactory portion of the dependent variable is explained by the exogenous variables. This is also supported by the test of predictive relevance (Q^2^), which assesses model fit [81]. The predictive relevance is indeed supported because the Q2 values shown in Table 4 are superior to zero [82].

The predictive quality is also evaluated via the global goodness of model fit, which has to be above 0.36 [79]. Indeed, our model’s global goodness of fit is 0.578, which shows acceptable global model validity.

#### 4.2.2. Hypotheses’ Verification

According to the findings illustrated in Table 4, H1a and H3b are rejected, as their *p*-values are greater than the required threshold of 5%. Accordingly, the convenience of the care process did not have a significant impact on trust in the service provider. Furthermore, physician concern was not found to be a significant determinant of service value. The findings also show that the convenience of the care process is a significant driver of service value for healthcare providers, with the following values (M = 0.221, t = 3.555, *p* = 0.01). Therefore, H1b is supported. Healthcare providers’ concerns were also found to be a significant predictor of trust toward the provider and service values for the following respective values (M = 0.326, t = 3.837, *p* = 0.000; M = 0.171, t = 2.314, *p* = 0.000). Therefore, H2a and H2b are supported. Physician concern was shown to be a significant driver of trust toward service providers for the following values (M = 0.281, t = 3.360, *p* = 0.001). Hence, H3a is accepted. Doctors’ reputation was found to be a significant predictor of both service values and trust toward the doctor for the following values (M = 0.339, t = 4.457, *p* = 0.000; M = 0.653, t = 16.105, *p* = 0.000). Hence, H4a and H4b are supported. Finally, the behavioral intentions of patients were found to be significantly predicted by trust toward the provider, trust toward the doctor, and service value for the following respective values (M = 0.319, t = 4.985, *p* = 0.000; M = 0.233, t = 2.742, *p* = 0.006; M = 0.310, t = 4.381, t = 0.000). Therefore, H5a, H5b, and H6 are supported.

The path parameters and their respective significance are shown in Table 4, which details the path coefficient, their respective *p*-values, and t-statistics.

**Figure 2:**
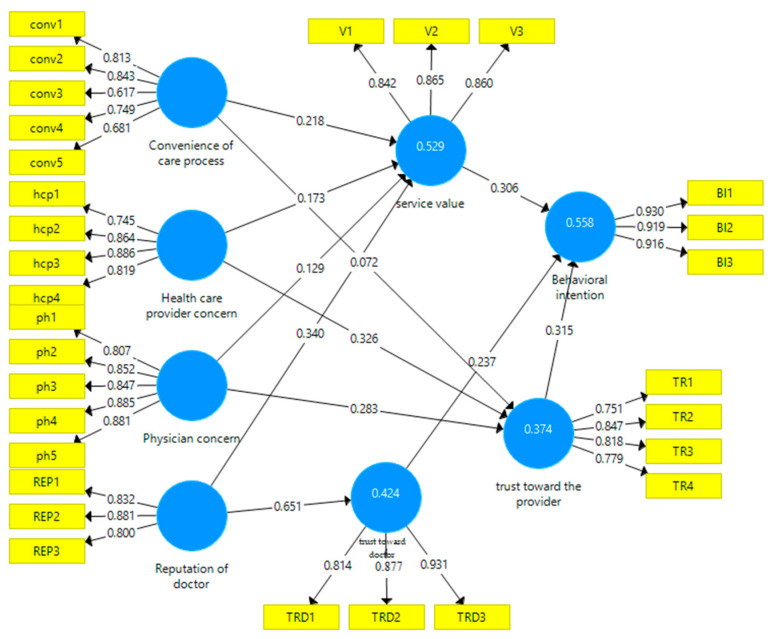
The measurement and structural model’s outputs (SmartPLS).

The outputs of the structural model from SmartPLS are shown in Figure 2.

## 5. Discussion

### 5.1. Hypotheses Discussion

Hypothesis H1a was rejected, which means that the convenience of the care process does not significantly influence trust in the healthcare provider. This result is not in line with prior studies which considered quality dimensions to be the primary factors leading to trust, like [79], Chang et al. [83], and Alrubaiee et al. [84]. Similarly, Han et al. [12] found that the interaction between physicians and patients is a vital building block of trust. However, our results could be explained by more holistic cues from clinics and hospitals. The measures of the convenience of the care process included items measuring the convenience and promptness of lab tests, payment procedures, appointments, and physician examinations. These elements of quality seem noninfluential when it comes to building a level of trust toward healthcare institutions. One important element that may have a higher influence is not included in the measurement scale: the reliability of the tests and examinations, which is likely a core element of healthcare outcomes. Another explanation is that initial trust can be placed in a doctor/hospital who/that has been recommended to the customer by relatives or friends whose advice will be trusted based upon the trust in the one who recommended, which adds more subjectivity to the customer evaluation.

In contrast, the two other dimensions of healthcare service quality, namely, healthcare provider concern (H2a) and physician concern (H3a), are significant predictors of trust toward the healthcare provider. This confirms the assumptions of the elaboration likelihood model [85] and leads us to conclude that primary factors or trust are the two dimensions of healthcare service quality.

The findings show that service value significantly depends on the convenience of the care process (H1b) and healthcare provider concern (H2b), which are two dimensions of healthcare service quality [4]. This is in line with the findings of previous studies on healthcare services [4] and other types of services linking quality with perceived value [48,86]. Indeed, the creation of value in this context is based, inter alia, on the convenience and promptness of the procedures to obtain services other than physician examination, such as lab tests, payment procedures, and setting up appointments. It also relies on interpersonal service quality elements, such as the friendliness and care of healthcare provider personnel.

The rejection of hypothesis H3b, which assumes a positive impact of the physician’s concern on service value, shows discordance with the findings of prior studies [4]. This discordance may be because Choi et al. [4] tested the impact of the whole concept of quality rather than each dimension, as in our study. The rejection of the hypothesis may also be explained by the categorization of physician concern as the core service, whereas service value is more about the work undertaken by the clinic and its administrative staff. Thus, we can infer the low importance of a physician’s contribution to the entire service value. Indeed, the patient may associate the healthcare organization’s service value with the whole process, including medicine, X-rays, lab results, etc. In addition, the empirical procedure did not identify a specific physician, knowing that generalists generally change in both private and public clinics.

The reputation of the doctor has a significant positive impact on service value (H4a). This is one of the research novelties of our study, as this link has been uncovered in previous studies. This path is explained by the fact that the reputation of a doctor reduces the risk of faulty examination or treatment. This risk is a psychological cost that the consumer may sacrifice if they are not sure about the doctor’s efficacy. Perceived value is defined as the ratio of output to sacrifice [50].

Similarly, a doctor’s reputation has a significantly positive impact on trust (H4b). This result is consistent with these two theories. The first is the elaboration likelihood theory, which considers reputation to be a secondary factor affecting trust [49,86]. The second is Spence’s signaling theory [18], which proposes that consumers take reputation as a signal to prove the trustworthiness of the provider. To the best of our knowledge, this path has not been covered in the field of healthcare services.

All the direct predictors of behavioral intention, namely healthcare provider (H5a), trust toward the doctor (H5b), and perceived service value (H6), have significant impacts on patients’ behavioral intentions. On the one hand, the significant impact of service value on behavioral intention is in line with prior research findings in healthcare research [4]. It confirms that the core variable leads to exchange values, even in critical services such as healthcare. On the other hand, the confirmation of the influence of both trust facets on behavioral intention is in line with the results of Zheng et al. [68], who were the first to consider hospital trust with doctor (interpersonal) trust. However, they found that doctors’ trust was greater than organizational trust, while we found the opposite. Another difference lies in the dependent variable. They considered purchase intentions, while our choice was more holistic because we used behavioral intentions. The outputs of the structural model from SmartPLS are shown in Figure 2.

### 5.2. Implications, Limitations, and Future Research Pathways

This research aimed to propose and test a conceptual model explaining the behavioral intentions toward healthcare providers by integrating the perceived service value, healthcare quality dimensions, trust toward the provider, and the doctor, as well as the reputation of the latter. The original model was then verified, and most of the hypotheses were confirmed.

Theoretically, the findings of this study show the importance of both facets of trust and value as important predictors of behavioral intentions toward a healthcare institution or provider. In fact, several studies in marketing have already approved the positive role of customers’ trust and service value in implementing the behavioral intention of their consumers [49,86]. However, from a healthcare perspective, this study is among the earliest of its kind to focus on drivers of patients’ behavioral intentions. In addition, this paper participates in the growth of theoretical knowledge as it is considered among the few studies that focus on verifying the impact of each element of healthcare service quality, thereby highlighting the facets of healthcare service quality that are more influential. Indeed, healthcare provider concerns and convenience of the care process are important sources of service value, while the provider’s trust is mainly influenced by the physician and the healthcare provider’s concerns. Furthermore, service value and both facets of trust significantly influence behavioral intentions. Additionally, this study is among the rare studies to examine the impact of physician reputation on trust in doctors. Lastly, it is among the earliest of its kind to investigate the relationships between patients and healthcare providers in a developing market like Saudi Arabia [37].

From a managerial perspective, these studies provide significant recommendations and insightful implications for practitioners in the healthcare sector to improve the behavioral intentions of their patients in both public and private clinics. First, the trust of patients in the healthcare provider needs to be enhanced by implementing more service-oriented personnel behavior: showing the care and friendliness of all contact employees, healthcare technicians, and physicians. Furthermore, patients who have stress, fear, and anxiety need the support of medical staff (doctors, nurses) by giving them more time to ease their concerns, providing them with the needed information about their illness and the options of their treatments, and ensuring them that they will do the necessary for a good recovery. Then, to reassure their patients, healthcare institutions should show their ability to provide them with quality, timely, and accurate service, which will reflect the image of honest, and performant service providers [87]. Second, doctors must ensure that the explanation of the medication process has been well understood by the patient and that coordination with other specialists handling the patient’s case is well established. These actions can help improve the level of trust and perception of value, which are necessary to trigger favorable behavioral intentions. Third, as the reputation of the doctor plays a significant role in the patient’s trust, hospitals are recommended to consider and communicate about doctors’ experience. Medical and ethical skills as well as interpersonal skills are vital criteria when hiring doctors [83]. Moreover, doctors are requested to be good listeners and ask the appropriate questions to understand the patient’s needs. The doctor is also required to be clear when asking questions by using simple language without using too much complex medical terminology. Also, to gain a good reputation, a doctor should be honest but also should offer hope even in a difficult situation. They should also be empathetic, collaborative, and curious in order to reduce the patient’s worries and fear. Lastly, managers of healthcare institutions are recommended to improve the quality of their services by adopting many measures like the digitalization of their services to make them more convenient, using new channels to communicate continuously with their patients (like social media) and showing them their concerns.

Several limitations of this research work should be highlighted. First, this study was conducted in only one developing country (Saudi Arabia), which may limit the generalizability of the results. Further research studies can be developed in other countries to compare their findings with those of the current study. Second, in this study, there is non-precision regarding the type of treatment required by the patients when they answered the survey. The results may change if specialist doctors are considered rather than generalists. Third, a further limitation of this research is the non-consideration of certain demographic factors such as education, income, marital status, and frequency of visits, mainly due to the intention to reduce the length of the survey. This study also omitted verifying the capacity of the survey respondents to visit the selected hospitals. Indeed, the structure of the sample, in terms of outdoor vs. in indoor patients, could have improved the replicability of the study. This could be an interesting intervention variable. Lastly, the type of healthcare provider, whether public or private, its size, and its offers (multiservice vs. one specialty) are expected to be important moderators leading to a change in our results. Thus, the researchers propose, as a plan for future studies, to integrate moderators such as the type of patient (outdoor vs. indoor patients), the specialty of the doctor, the type of healthcare provider, and its size or multi-specialization. The interaction of both types of trust was not examined in this study. We propose the integration of this concept into future investigations.

## 6. Conclusions

The healthcare market, like all other markets, becomes highly competitive, and to gain a competitive advantage, hospitals are required to build long-term relationships with their patients. Therefore, a better understanding of patient behavior is crucial. This study was developed in this context and investigates the main drivers of the behavioral intentions of patients. The findings showed that service quality with its three dimensions (convenience, services provider concern, and physician concern) positively influences patients’ trust in service and service value. The reputation of doctors positively influences their trust in doctors. The trust of patients and service value were found to be interesting drivers of patients’ behavioral intentions. These results highlight the need to improve the service quality and value of healthcare institutions as well as enhance doctors’ reputations. Hence, to survive and grow in a highly competitive healthcare market, organizations should consider the patients as "customers" and understand their behavior as a major priority for building long-term relationships with them to win the battle of competitiveness.

## Figures and Tables

**Table 1:** Sample demographic characteristics (N = 242).

Variable	Classification	Number	Percentage
Gender	Male	125	51.65%
	Female	117	48.34%
Age range	18–25	21	8.67%
	26–30	83	34.30%
	31–40	93	38.42%
	41–50	34	14.04%
	More than 50	11	4.55%
Nationality	Saudi	219	90.5%
	Non-Saudi	23	9.5%
Public vs. private hospital	Public hospital	92	38%
	Private hospital	150	62%

**Table 2:** Composite reliability and Cronbach’s alpha.

Construct	Cronbach’s Alpha	Rho_A	CR	(AVE)
Behavioral intention	0.911	0.911	0.944	0.849
Convenience of the care process	0.799	0.826	0.861	0.555
Healthcare provider concern	0.848	0.853	0.898	0.689
Physician concern	0.908	0.911	0.931	0.731
Reputation of doctor	0.789	0.795	0.876	0.703
service value	0.819	0.833	0.891	0.732
Trust toward doctor	0.846	0.858	0.907	0.766
Trust toward the provider	0.814	0.823	0.876	0.639

Note. CR: Composite reliability; AVE: Average variance extracted.

**Table 3:** Predictive relevance indicators.

Variable	Classification	F2	R2	Q2
Behavioral intention	Service Value	0.111	0.558	0.442
	Trust in the physician	0.049		
	Trust toward the HC provider	0.113		
Trust toward the HC provider	Convenience	0.004	0.374	0.303
	HC provider concern	0.07		
	Physician concern	0.065		
Trust in the physician	Reputation of the doctor	0.735	0.424	0.303
Service value	Convenience	0.053	0.529	0.358
	HC provider concern	0.025		
	Physician concern	0.014		
	Reputation of the doctor	0.115		

**Table 4:** Path coefficient of the research hypotheses.

	Original Sample (O)	Sample Mean (M)	Standard Deviation (STDEV)	t-Statistics (|O/STDEV|)	*p*-Values	Hypotheses
Convenience of care process → Trust toward the provider	0.072	0.079	0.072	1.008	0.314	H1a rejected
Convenience of care process → Service value	0.218	0.221	0.061	3.559	0.000	H1b accepted
Healthcare provider concern → Trust toward the provider	0.326	0.326	0.085	3.837	0.000	H2a accepted
Healthcare provider concern → Service value	0.173	0.171	0.075	2.314	0.021	H2b accepted
Physician concern → Trust toward the provider	0.283	0.281	0.084	3.360	0.001	H3a accepted
Physician concern → Service value	0.129	0.131	0.086	1.499	0.134	H3b rejected
Reputation of doctor → Service value	0.340	0.339	0.076	4.457	0.000	H4a accepted
Reputation of the doctor → Trust toward the doctor	0.651	0.653	0.040	16.105	0.000	H4b accepted
Trust toward the provider → Behavioral intention	0.315	0.319	0.063	4.985	0.000	H5a accepted
Trust toward doctor → Behavioral intention	0.237	0.233	0.086	2.742	0.006	H5b accepted
Service value → Behavioral intention	0.306	0.310	0.070	4.381	0.000	H6 accepted

## Data Availability

The data that support the findings of this study are available from the corresponding author, [Zohra Ghali], upon reasonable request.

## Appendix A

**Table A1:** Constructs measurement items.

Construct		Items	Source
The convenience of the care process	1	The procedure to get the lab test was convenient	Choi et al. [4]
	2	The lab test was done in a prompt way.	
	3	The payment procedure was quick and simple.	
	4	The process for setting up the appointment was simple and easy.	
	5	I did not have to wait long for the medical examination from the physician.	
Healthcare providers’ concern	1	The nurses were friendly	Choi et al. [4]
	2	The care providers explained the medication process well.	
	3	Care providers truly cared for me	
	4	There was good coordination among the care providers	
Physician’s concern	1	The physician was polite	Choi et al. [4]
	2	The physician adequately explained my condition, examination results, and the treatment process	
	3	The physician allowed me to ask many questions enough to clarify everything	
	4	The physician paid enough consideration to my concerns in deciding on a medical procedure	
	5	The physician made me feel comfortable	
Reputation of the doctor	1	My doctor always fulfills his commitments	Torres et al. [65]
	2	My doctor has a good reputation	
	3	My doctor’s reputation is better than the reputation of other Doctors	
Service value	1	Compared with the time and money that has been given the service was valuable	Zeithaml [50]
	2	At a price has been paid, the service was acceptable (excellent compared to the price)	
	3	It was worth taking this healthcare service rather than others	
Behavioral intention	1	I will recommend that other people use this hospital	Choi et al. [4]
	2	If I needed medical services in the future, I would consider this hospital as my first	
	3	I will tell other people good things about this hospital	
Service provider’s trust	1	I often hear positive news about the hospital in the media (on TV, the internet, and in magazines)	Zheng et al. [68]
	2	I hear the hospital is recognized by official authority and/or the National Institutes of Health	
	3	I hear the hospital gains accreditation recognized by government official authority	
	4	I trust my doctor’s decisions about which treatments are best for me	
Trust in Doctors	1	My doctor only thinks about what is best for me	Zheng et al. [68]
	2	I have no worries about putting my life in my doctor’s hands	
	3	All in all, I have complete trust in my doctor	

**Table A2:** Item cross-loadings.

	Behavioral Intention	Convenience of the Care Process	Healthcare Provider Concern	Physician Concern	Reputation of Doctor	Service Value	Trust toward Doctor	Trust toward the Provider
BI1	0.930	0.539	0.517	0.587	0.610	0.612	0.614	0.602
BI2	0.919	0.573	0.522	0.519	0.590	0.596	0.615	0.590
BI3	0.916	0.572	0.538	0.565	0.550	0.566	0.608	0.602
REP1	0.537	0.471	0.610	0.692	0.832	0.593	0.584	0.486
REP2	0.568	0.435	0.508	0.608	0.881	0.509	0.582	0.525
REP3	0.484	0.360	0.384	0.457	0.800	0.517	0.460	0.475
TR1	0.539	0.343	0.365	0.319	0.404	0.375	0.476	0.751
TR2	0.462	0.323	0.458	0.395	0.465	0.393	0.502	0.847
TR3	0.452	0.292	0.358	0.372	0.387	0.402	0.477	0.818
TR4	0.587	0.441	0.584	0.602	0.584	0.551	0.705	0.779
TRD1	0.514	0.388	0.498	0.552	0.535	0.491	0.814	0.592
TRD2	0.586	0.514	0.581	0.568	0.535	0.631	0.877	0.591
TRD3	0.638	0.485	0.586	0.589	0.633	0.659	0.931	0.641
V1	0.474	0.465	0.499	0.454	0.504	0.842	0.523	0.455
V2	0.488	0.491	0.518	0.440	0.503	0.865	0.596	0.453
V3	0.657	0.521	0.549	0.639	0.631	0.860	0.621	0.503
conv1	0.532	0.813	0.577	0.500	0.439	0.506	0.510	0.447
conv2	0.553	0.843	0.570	0.470	0.413	0.495	0.443	0.393
conv3	0.330	0.617	0.343	0.341	0.337	0.315	0.256	0.230
conv4	0.405	0.749	0.440	0.390	0.367	0.386	0.337	0.265
conv5	0.399	0.681	0.519	0.354	0.321	0.410	0.370	0.281
hcp1	0.495	0.571	0.745	0.601	0.462	0.510	0.466	0.447
hcp2	0.495	0.498	0.864	0.598	0.541	0.494	0.542	0.484
hcp3	0.494	0.566	0.886	0.617	0.556	0.536	0.609	0.540
hcp4	0.404	0.593	0.819	0.455	0.440	0.492	0.481	0.409
ph1	0.516	0.437	0.514	0.807	0.568	0.479	0.538	0.416
ph2	0.556	0.529	0.671	0.852	0.638	0.530	0.625	0.479
ph3	0.481	0.431	0.552	0.847	0.578	0.504	0.476	0.444
ph4	0.502	0.488	0.566	0.885	0.621	0.531	0.524	0.506
ph5	0.529	0.501	0.628	0.881	0.612	0.558	0.614	0.492
